# Psychopathological Determinants of Quality of Life in People with Borderline Personality Disorder

**DOI:** 10.3390/jcm12010030

**Published:** 2022-12-20

**Authors:** Pentagiotissa Stefanatou, Lida Alkisti Xenaki, George Konstantakopoulos, Anthoula Papaiakovou, Irene Ralli, Aristea D. Berk, Diamantina S. Katopodi, Aphrodite D. Pantagoutsou, Aimilia Charitaki, Maria Ginieri-Coccossis, Eleni Giannouli, Ioannis A. Malogiannis

**Affiliations:** First Department of Psychiatry, Eginition Hospital, School of Medicine, National and Kapodistrian University of Athens, 11528 Athens, Greece

**Keywords:** borderline personality disorder, quality of life, WHOQOL-BREF, depression, suicide, state anxiety, trait anxiety, personality disorders, comorbidity, disease severity

## Abstract

Background: Subjective quality of life (SQOL) in people with borderline personality disorder (BPD) is a marker of disease burden; a crucial treatment outcome; an indicator of psychosocial functioning; and a measure of interventions’ effectiveness. Given the dearth of consolidated data, the current study examined psychopathological determinants of global and domain-specific SQOL in people with BPD. Methods: Hierarchical regression models were employed to examine in BPD patients (*n* = 150) the relationships of the number of BPD diagnostic criteria; the co-occurrence of other personality disorders (PDs); depression; state and trait anxiety; suicidality; self-harming; alcohol and substance use disorders with SQOL indices, namely physical health, psychological health, social relationships, environment, overall QOL and overall health. SQOL was estimated using the WHOQOL-BREF instrument. Results: Co-existing symptomatology such as depression, state and trait anxiety, and personality pathology, namely the co-occurrence of other PDs, exhibited significant associations with global and domain-specific SQOL, albeit depression was the strongest determinant of the most SQOL domains. In contrast, the number of BPD diagnostic criteria and central illness features such as suicidality, self-harming behaviour, and impulsivity manifested through alcohol and substance use did not exhibit significant associations with any SQOL dimension. Conclusions: Comprehensive assessment of depressive symptoms should be regularly implemented in BPD services to facilitate early detection and treatment, thereby ensuring patients’ SQOL. Accordingly, tackling anxiety and other PDs co-occurrence through appropriate interventions can facilitate more effectively SQOL improvement. Our findings can be explained by the hypothesis that co-existing psychopathology such as depression, anxiety and co-occurrence of other PDs in BPD patients represent illness severity indices rather than comorbid disorders, and might fully mediate the effect of BPD traits on SQOL. Future mediation analysis is required to elucidate this hypothesis.

## 1. Introduction

Borderline Personality Disorder (BPD) is the fourth most prevalent among the ten DSM-5 personality disorders (PDs), affecting 1–2% of the general population, with a high prevalence (15–40%) in the clinical setting [1]. The onset of symptoms typically occurs during adolescence and young adulthood, subsiding later in life [2]. BPD is predominantly characterized by an unstable sense of self and others, fear of abandonment, volatile emotionality, affective and behavioural dysregulation and difficulties in impulse control, which are associated with high rates of suicide [3,4,5,6]. In addition, BPD is frequently accompanied by other mental syndromes, such as anxiety, depression, substance use, and other PDs [7,8,9]. The severe morbidity along with the increased sensitivity to perceived interpersonal frustration pose a marked therapeutic challenge, so BPD patients can be considered difficult to treat among other psychiatric populations [1,10,11]. Moreover, this multifaceted clinical picture has pragmatic and personal repercussions in terms of profoundly impaired functionality and quality of life (QOL) [12,13,14]. Indeed, BPD patients have difficulties in sustaining enduring emotional relationships, successful vocational records and satisfactory income [13,15,16], while their own reports regarding mental, social and physical well-being are remarkably negative [14,17,18,19].

The psychopathology of BPD is hypothesized to be grounded on a combination of inherent emotional dysregulation and invalidating early experiences from significant others [3,20], resulting in pervasive maladaptive traits that are reflected on clinical manifestations, refractory to pharmacotherapy [21]. Different psychotherapeutic approaches have been developed [transference-focused psychotherapy (TFP), mentalization-based treatment (MBT), schema therapy (ST), and dialectical behaviour therapy (DBT)] with beneficial results in terms of symptomatic remission of BPD [22]. Improvement in psychosocial functioning and QOL is still detectable, but to a substantially lesser extent, which means that symptom reduction is not equivalent to restoration of QOL to normal levels [23] and patients’ needs seem to be suspended therapeutically [24].

Traditionally in clinical practice, assessment of the course of a mental disorder was followed mainly through symptoms’ amelioration and rates of remission. However, in the past decade, outcome assessment in mental diseases has broadened to incorporate a recovery focus. Active user participation in the rehabilitation process as well as incorporating user perspective on outcomes via patient-reported measures are critical components of recovery-oriented clinical practice and research in mental disorders [25]. The recovery approach emphasizes subjective quality of life (SQOL), independent living in the community, educational and vocational attainment, satisfying interpersonal and social relationships, social inclusion, and a meaningful life [26]. In this regard, SQOL is considered to be a critical therapeutic outcome, and thus the touchstone for an effective, recovery-focused treatment of mental diseases [26,27] including PDs [28,29]. SQOL has been conceptually linked to “life satisfaction”, “happiness with life” or “subjective well-being” [30]. The World Health Organization (WHO) defines SQOL as “individuals’ perceptions of their position in life in the context of the culture and value systems in which they live and in relation to their goals, expectations, standards and concerns”. WHO considers QOL as an indicator of the burden of the disease. More specifically, disease burden has been also characterized as loss of welfare, subjective well-being/quality of life [31]. In this respect, WHO views health-related SQOL as a measure of the perceived impact of the disease on major life domains [32].

Focusing on BPD, SQOL represents a main marker of disease burden and an important aspect of clinical and psychosocial outcomes [14,22,33,34]. In the context of recovery-oriented treatment of mental illnesses, it has been suggested that broadening the focus of psychotherapy for BPD by explicitly including SQOL as a therapy goal may improve the psychosocial functioning of patients with BPD, as well as their long-term recovery after therapy [35]. In this respect, improvement in BPD patients’ perceptions of their health in relation to their QOL is considered a main goal of healthcare and a crucial treatment outcome [14]. Concurrently, SQOL has emerged as a crucial indicator of the effectiveness of therapeutic interventions. Specifically, it has been recommended that the evaluation criteria of treatment efficacy in BPD should not be limited to the amelioration of central BPD traits and co-existing symptomatology, but should also extend to the improvement of SQOL [14,23]. According to previous evidence, SQOL is linked to both treatment-seeking behaviour and treatment adherence [34]. Moreover, reduced SQOL across physical, psychological, and social life domains is indicative of impaired functioning in the respective areas [14]. Notably, functional deficits in the psychosocial and physical domains in BPD patients are related with failure to achieve or sustain recovery after therapy [36]. In light of the above, the evaluation of both global and domain-specific SQOL as well as the identification of the factors that impair or improve SQOL are of utmost importance for the attainment of major recovery outcomes in BPD. However, research in the field is limited.

Based on a prior review study, impairment of SQOL in BPD is linked to the severity of the disorder’s psychopathology, while reducing illness severity could potentially enhance SQOL [14]. However, recent research shows that SQOL improvement as a consequence of therapy for BPD may not just be a result of symptom reduction [22]. Despite the relative dearth of consolidated data, previous studies have found that SQOL is considerably reduced in BPD patients compared to non-clinical populations [19,23]. The presence of an increasing number of DSM diagnostic criteria for BPD is correlated with a more severe disorder [1]. Even the presence of a single BPD criterion has been strongly related with more concomitant mental diseases, current suicidal ideation, prior suicide attempts, previous hospital admissions, and impaired functioning. However, it should be noted that although the number of BPD traits has emerged as a significant indicator of illness severity in people with subthreshold BPD features and in patients who meet the DSM criteria for BPD, it proved to be a stronger indicator of disease severity for the former compared to the latter [37]. In prior reports, an increase in the number of BPD diagnostic criteria has been linked to worse global SQOL in youths with BPD traits [38] and in veterans with BPD [39]. Moreover, illness severity in BPD is also reflected through comorbidity with other mental disorders, including the co-existence of other PDs. Specifically, Cramer et al. [33] found that BPD was among the PDs with the lowest global and domain-specific SQOL in a population with diverse PDs, with the data also indicating that the higher the number of PDs as well as the number of PD traits, the worse the SQOL in BPD patients. However, another study suggested that BPD is not the most exacerbating PD in terms of QOL and that the number of PD diagnoses, not the type, is associated with declines in QOL [34]. Additionally, in a sample of adolescents with personality pathology, no specific personality disorder diagnosis significantly predicted an impaired QOL [40]. In a longitudinal study investigating the differential impact of the various clusters of PDs on SQOL [41], antisocial PD and BPD were associated with poorer SQOL in the physical health domain.

Suicidality and self-harming behaviour are considered to be central illness features in BPD [3,11] and have also been speculated to negatively affect the SQOL of BPD patients [14], particularly in the physical health domain [41]. However, no studies so far have examined the independent contribution of suicidality and self-harming behaviour to SQOL in an adult population of BPD patients.

Regarding coexisting symptomatology, in various large patient samples, the lifetime rates of major depressive disorder vary from 61 to 83%, while the lifetime rate of anxiety disorders is 88% in patients with BPD [1]. Importantly, patients with BPD and concomitant major depressive disorder present more severe deficits in psychosocial functioning and higher rates of suicide attempts [1]. In prior reports, increasing levels of depression exhibited the strongest associations with decreasing SQOL in BPD patients [19,23] and in youths with BPD traits [38]. However, in the last two studies, only global SQOL was investigated, not SQOL domains. Anxiety has been also assumed to adversely impact SQOL in BPD [14]. Grambal et al. [19] explored the effect of anxiety on global and domain-specific SQOL in a population of BPD patients with comorbid anxiety disorders and they found that the level of anxiety was associated with SQOL in bivariate analysis, but not in the following multiple regression analysis. Furthermore, in another report [42], BPD patients with comorbid post-traumatic stress disorder (PTSD) had considerably worse SQOL than those with BPD or PTSD alone.

One of the common syndromes observed in BPD patients that has been hypothesized to have a detrimental effect on SQOL is substance use disorders [14]. In a study investigating the relationship between SQOL and PDs among injecting drug users, BPD was the most prevalent PD, and BPD drug users exhibited poorer SQOL in psychological health and environmental domains [43]. To our knowledge, so far, the independent effects of alcohol and substance use on the SQOL of patients with a primary BPD diagnosis have not yet been investigated.

Concerning sociodemographic factors, previous findings are inconclusive regarding gender, with some authors reporting that female gender is associated with worse SQOL in BPD [44], while others did not find sex-related differences [38]. Older patients were found to have poorer SQOL [45,46,47], despite reductions in symptoms over time [1].

In conclusion, it appears that the evidence on the relationship between psychopathology and SQOL in BPD patients is rather limited and inconclusive. Specifically, prior research has not consistently identified the aspects of BPD psychopathology, including associated psychopathological manifestations that are most directly accountable for the deterioration of global and domain-specific SQOL. This is likely attributable to a variety of limitations in earlier research. First, only a few studies seeking determinants of SQOL have focused on an adult clinical population with a primary or single BPD diagnosis [19,23,46,48]. Second, most of the prior research studied just a subset of the main BPD traits and co-existing symptoms, making it hard to identify those that have the greatest influence on SQOL. Third, the majority of studies on patients with a primary BPD diagnosis assessed global SQOL predictors rather than domain-specific elements [23,48]. In the study of Frías et al. [46], which examined domain-specific SQOL, only the effects of age on psychopathology and SQOL were tested. Given that SQOL is a marker of disease burden and thus a crucial clinical outcome [33,34]; an indicator of psychosocial functioning [14,22,46]; and a measure of the effectiveness of treatment interventions [22,23], it is important to find the psychopathological determinants of SQOL in BPD. Moreover, key life domains of SQOL should be extensively studied as they could be differentially impaired. Identifying the mental health factors that impair global and domain-specific SQOL will inform the design of tailor-made interventions to address them, and therefore, it is expected to improve SQOL and achieve crucial recovery outcomes.

Against this background, in the current study, we examined the relationships of the main BPD psychopathological dimensions, including common co-existing symptoms such as depression and anxiety as well as other personality pathologies, with global and domain-specific SQOL in BPD patients. Due to the paucity of prior evidence, the aim of the study was to figure out thoroughly which aspects of psychopathology in BPD patients have the strongest independent associations with SQOL impairments, controlling for sociodemographic variables. Specifically, we explored whether essential BPD features and other personality pathologies, namely (1) the number of BPD criteria, (2) the occurrence of additional PD(s), (3) self-harming behaviour, (4) suicidality and (5) impulsivity manifested through alcohol and substance use are significantly associated with SQOL indices, including physical health, psychological health, social relationships, environment, overall QOL, and overall health. Based on previous evidence indicating that the number of BPD criteria and the occurrence of additional PD(s) have been associated with compromised SQOL in populations with BPD traits [33,38,39], we hypothesized that, in the current study, both these severity indices would be negatively linked with SQOL domains. Furthermore, we examined whether depression and anxiety are strongly associated with the aforementioned SQOL indices, above and beyond personality pathology. We employed both state and trait anxiety as predictor variables in order to explore whether anxiety intensity (state) and anxiety frequency (trait) are differentially associated with global and domain-specific SQOL. Considering that (a) depression and anxiety in BPD patients are associated with detrimental effects such as compromised psychosocial and vocational functioning [1]; and (b) the few previous studies investigating SQOL in BPD found that depression was the strongest determinant of SQOL decline in patients with BPD and youths with BPD traits [19,23,38], we anticipated that in the current investigation, depression and anxiety would be more strongly related to SQOL in BPD patients, above and beyond essential BPD features, the number of BPD criteria, and the co-occurrence of other PD(s). Furthermore, we hypothesized that depression and anxiety might have a mediating role in the relationship between essential BPD features as well as the number of BPD criteria and SQOL, both global and domain specific.

It is of note that the cross-sectional study design cannot support strong causal inferences or temporal ordering between the predictor variables of BPD psychopathology and the SQOL outcomes. Our cross-sectional data may be sensitive to reverse causality, i.e., poor SQOL may also negatively affect dimensions of mental health in BPD patients. Our findings, however, will be interpreted in terms of the predictive variables as potential determinants of global and domain-specific SQOL, as was the case in all previous cross-sectional studies in the field [19,23,38,46,48], since the emerging relationships may provide crucial insights into the putative effects of BPD psychopathological dimensions on SQOL, the causality and direction of which could be tested in future longitudinal studies.

## 2. Materials and Methods

### 2.1. Study Design and Participants

The study took place at Personality Disorders specific sector of the First Department of Psychiatry at Eginition Hospital, School of Medicine, National and Kapodistrian University of Athens. A Specialized Psychotherapeutic Program operates in the Personality Disorders sector which was founded in 1999 [11,49]. This includes an outpatient service as well as a Day Hospital. It provides group and individual psychoanalytic psychotherapy to patients with borderline personality organization [50] and severe personality disorders [51], in combination with other treatment practices (psychiatric consultation, pharmacotherapy and hospitalization, if indicated, as well as psychodynamic art psychotherapy and family therapy) [11,52]. Participants were help-seeking adults, 18–56 years of age, who met the DSM-5 diagnostic criteria for BPD [4]. Exclusion criteria were: (a) lifetime diagnosis of a psychotic disorder, (b) presence of acute psychopathology requiring urgent psychiatric treatment, (c) presence of autism spectrum disorder, (d) history of organic brain disorder, and (e) intellectual disability (IQ < 70). Subjects were referred to our service by other mental health services or through self-referral and were enrolled consecutively after basic clinical assessment. Evaluation was performed by two psychiatrists and four clinical psychologists specially trained in the performance of the specific clinical interviews. All interviews were conducted in person in the outpatient service before patients received treatment. The study combined baseline diagnostic, demographic, and functioning data. During a time period of three years (from 2018 to 2021), we reached a sample of 150 adults.

### 2.2. Assessment Tools

Subjects participated in detailed demographic and clinical assessment of the BPD psychopathology and co-existing clinical entities. To exclude intellectual disability, we applied the Greek version of the Wechsler Adult Intelligence Scale, fourth edition (WAIS-IV^GR^) [53,54], before enrolment in the study. For the evaluation of participants’ present psychopathology and lifetime history of psychiatric disorders, the Greek version of the semi-structured interview Mini International Psychiatric Interview (MINI) [55,56,57] was used. To establish the diagnosis of BPD and to assess the number of BPD traits as well as other PD diagnosis, the Greek adaptation of the Structured Clinical Interview for DSM-5 Personality Disorders (SCID-5-PD) [58,59] was applied. This is a semi-structured interview specifically designed to assess the DSM-5 Section II PDs, providing both dimensional (i.e., the sum of sub-clinical and clinical scores) and categorical diagnoses. The “5-year rule” was followed, which means that the ratings correspond to a behaviour typical of the past 5 years. Self-harming behaviour was assessed as a unique psychopathological feature by the Self-Harm Inventory (SHI) [60,61]. This is a 22-item self-report questionnaire with a dichotomous answer (yes or no), exploring a range of lifetime history of self-destructive behaviours and thoughts. The SHI total score is the simple sum of “yes” responses, with a maximum possible score of 22. Higher scores reflect more serious self-harm. The internal consistency value for the SHI was satisfactory (Cronbach’s α = 0.71). Suicide risk was assessed by the Greek adaptation of the Suicide Risk (SR) scale [62,63]. This is a self-applied scale consisting of 15 question-items with a dichotomous answer (yes or no). The scale explores factors such as suicidal ideation, negative feelings towards self and others, hopelessness, depression and other, yielding a maximum score for suicide risk of 15 points. Cronbach’s α for the SR scale was 0.84.

Depressive symptoms were assessed with the Greek version of the Beck Depression Inventory–II (BDI-II) [64,65]. This is a self-report 21-item questionnaire scored on a four-point (0–3) Likert scale. The minimum and maximum scores are 0 and 63, respectively. Cronbach’s α for the BDI II was 0.85. For the evaluation of current anxiety state and the trait aspects of “anxiety proneness” we used the State-Trait Anxiety Inventory (STAI) [66,67]. This is self-report psychometric tool with 2 subscales: (a) the State Anxiety Scale (S-Anxiety) measuring the present intensity of symptoms, and (b) the Trait Anxiety Scale (T-Anxiety) measuring the frequency of feelings. Each subscale has 20 items. Item scores should be added to obtain subtotal scores, ranging from 20 to 80, with a higher score indicating higher anxiety. Cronbach’s α for the State subscale was 0.93 and for the Trait subscale it was 0.92. As to participants’ quality of life, the Greek version of the World Health Organization Quality of Life Instrument Short Form (WHOQOL-BREF) [68,69] was used. WHOQOL-BREF consists of 26 questions and is divided into four domains: Physical health and Level of Independence, Psychological health and Spirituality, Social relationships, and Environment. The physical health domain addresses issues related to functional capacity, energy levels, pain load, daily activities, and pattern of sleep. The psychological health domain includes factors regarding self-representation, positive and negative thinking, self-esteem, cognitive abilities and general mental condition. The social relationships domain involves the context of interpersonal relationships between individuals and/or groups in the social milieu in which a person lives. The environmental domain stands for the natural and living environment that a person inhabits and experiences as his/her everyday milieu. The environmental domain covers issues related to housing conditions, population density, transportation, recreation, physical environment (e.g., pollution, grime, and noise), financial resources, safety, access to health and social services, and opportunities to acquire new skills and knowledge. The Greek version has four additional items for cultural adaptation purposes, involving nutrition, social life, family life, and job satisfaction. Assessment is performed at a 5-point scale (very bad, bad, neutral, good, very good). Additionally, two items are examined separately: question 1 which addresses the individual’s perception of global QOL, and question 2, which addresses his/her overall health perception. Internal consistency values (Cronbach’s α) for the Physical health, Psychological health, Social relationships, and Environment domains were 0.81, 0.79, 0.76, 0.67 respectively, whereas for the overall QOL/overall health items it was 0.89.

### 2.3. Data Analysis

The responses of all participants who matched the inclusion criteria were included in the analysis. WHOQOL-BREF subscales were operationalized as continuous variables. The number of BPD criteria, additional PD diagnosis, BDI-II score, STAI-State and Trait score, self-harm inventory score, and suicide risk scale score were also operationalized as continuous variables, while alcohol and substance use disorder (AUD/SUD) were operationalized as a binary variable. The sociodemographic variables that were used included gender, age, and educational level.

We built hierarchical linear regression models to account for the relation of: (a) Number of BPD diagnostic criteria, (b) Additional PD diagnosis, (c) Self-harming behaviour, (d) Suicide Risk and (e) Alcohol and Substance use disorder, (f) depression and (g) state and trait anxiety with WHOQOL-BREF subscales, after adjusting for sociodemographic variables as potential confounding factors. We employed all socio-demographic characteristics as block 1 variables; the Number of BPD criteria, Additional PD diagnosis and the essential BPD features such as Self-Harming behaviour, Suicide Risk, and impulsivity manifested through Alcohol and Substance use disorder (AUD/SUD) were entered as block 2 variables, whereas common co-existing BPD symptomatology such as depression and state and trait anxiety as estimated by the BDI-II and STAI-State and STAI-Trait, respectively, were employed as block 3 variables. Six separate analyses were conducted, addressing each subscale of the WHOQOL-BREF as the dependent variable, namely physical health, psychological health, social relationships, environment, overall QOL, and overall health. *p*-values less than 0.05 were considered significant. All statistical analyses were carried out using IBM SPSS version 28. Prior to conducting each hierarchical multiple regression, the relevant assumptions were tested. A sample size of 150 was deemed adequate given eleven independent variables to be included in each of the above models. For regression equations using six or more predictors, an absolute minimum of 10 participants per predictor variable is appropriate [70]. According to our power analysis, a group of 123 participants would enable us to detect associations with medium effect size (f^2^ = 0.15) between 11 predictors and the dependent variable in each of our multiple regression models with 80% power at *p* < 0.05. Collinearity statistics were within accepted limits in all regressions, as the variance inflation factors (VIFs) ranged from 1.01 to 2.14 across models.

## 3. Results

Our sample consisted of 150 BPD subjects. Demographic (gender, age, education level, family status, employment, living area) and clinical characteristics (number of BPD criteria, additional PD diagnosis, suicide risk and self-harming scores, number of hospitalizations, BDI-II, STAI-State and STAI-Trait scores, affective, anxiety, substance use and other disorders, as well as dimensions of WHOQOL-BREF) are presented in Table 1.

Table 2 displays the hierarchical regression models predicting WHOQOL-BREF dimensions. In sum, our results indicated that in subjects with BPD, the severity of coexisting depressive symptoms negatively affects all domains of WHOQOL-BREF except environment, additional PD diagnosis decreases the level of physical and psychological health, and higher intensity and frequency of anxiety aggravates psychological health, with increased frequency negatively affecting overall health as well.

Regarding Physical Health, after adding all three steps to the hierarchical model, additional PD diagnosis and BDI-II were significantly negatively associated, with an R-square value of 0.256 (25.6% of the variance accounted by all variables in the model). As for Psychological Health, the predictors that remained statistically significant through the final model were again additional PD diagnosis, BDI II, State and Trait anxiety that had a negative impact on psychological aspects of the quality of life of BPD patients. In fact, this association was the strongest with an R-square value of 0.354 (35.4% of the variance accounted by all variables in the model). Concerning Social Relationships, age was found to be negatively correlated, and, again, the severity of coexisting depressive symptoms had a statistically significant negative association. Nevertheless, the R-square was 0.165, representing a relatively small proportion of the variance for the dependent variable explained by all independent variables in the model. In regard to Environment, no statistically significant association was found between any potential predictor and the environmental domain of WHOQOL-BREF, with 6.1% of the variance accounted for by all variables in the model. Regarding Overall QOL, only increased severity of depressive symptoms was significantly associated in a negative way, with an R-square value of 0.118, which can be interpreted as all variables accounting for 11.8% of the variance in the overall QOL scores. Finally, regarding Overall Health, increased level of education was found to be a significant positive predictor variable, while the increased frequency of anxiety symptoms appeared to have a significant negative impact. The R-square value of the final model was 0.221, which can be interpreted as the included independent variables accounting for 22.1% of the variance in Overall Health.

## 4. Discussion

The current study examined connections between the main BPD features, prevalent co-existing symptoms, and other personality pathologies with global and domain-specific SQOL, aiming to identify those psychopathological variables most directly involved in SQOL deterioration. To our knowledge, this is the first study that thoroughly investigated psychopathological determinants of global and domain-specific SQOL in adult patients with a primary or single BPD diagnosis. The main findings of our study highlight the pivotal role of co-existing symptoms and other personality pathologies on the SQOL of BPD patients. Specifically, our findings indicate that co-existing symptoms of depression and anxiety are strongly associated with SQOL decline in BPD patients, above and beyond the essential BPD features, the number of BPD diagnostic criteria, and additional PD(s) occurrence, confirming the main hypothesis of the present study. That is, the number of BPD diagnostic criteria, suicidality, self-harming behaviour, alcohol and substance use disorders had no significant connection with any SQOL dimension, above and beyond depression and anxiety. Importantly, depression was strongly associated with more SQOL domains compared to all other variables, in particular physical health, psychological health, social relationships, and overall QOL. The occurrence of additional PD(s) was significantly associated with physical and psychological SQOL domains but not above and beyond depression and anxiety. Finally, older age was associated with a decreasing SQOL in social relationships, while more academic years were related to higher perceived overall health.

Although there have been a few prior studies evaluating SQOL in BPD populations, a comparative analysis of the findings is conceivable but limited due to the discrepancies in SQOL measures and indices; study methods and aims; participants’ clinical condition; and age categories.

In the current study, the strong associations of depression with SQOL in the most key life domains suggest that the severity of depression is a potential predictor of SQOL outcome in BPD. However, longitudinal studies are needed to confirm this hypothesis. Our findings accord with two previous studies on BPD patients [19,23], as well as with a report on youths with BPD traits [38]. In particular, the substantial associations we revealed between depression and physical, psychological, social, and global SQOL are consistent with the results of Grambal et al. [19], who found depression to be inversely related to physical, social, occupational, as well as global SQOL. This is the only prior study that investigated both global and domain-specific SQOL focusing on BPD patients, albeit all participants were inpatients with a comorbid anxiety disorder, whereas a different SQOL tool was employed. Additionally, in that study, as Grambal et al. mention [19], it is not clear whether the burden on SQOL was due to BPD psychopathology or that of anxiety disorders. In the study by Guillén et al. [23], depression and resilience were both major predictors of a single global SQOL measure in BPD patients, yet depression was the only psychopathological variable evaluated. Thompson et al. [38] also found that depression was the strongest independent predictor of SQOL in youths with BPD traits. Again, the investigation was confined to a global SQOL outcome, while the assessment of SQOL psychopathological determinants was restricted to a very small number of variables, possibly owing to a different population (youths with a mean number of traits subthreshold for BPD diagnosis). We found an inverse relationship between trait and state anxiety and SQOL in psychological health and overall health, in contrast to previous studies in patients with BPD [19] and youths with BPD traits [38]. This might be due to the essential difference between study samples; all participants in the study by Grambal et al. [19] were BPD patients with comorbid anxiety disorders and this may have decreased the variance in anxiety levels, whereas many participants with subthreshold BPD features were included in the study by Thompson et al. [38].

The negative relationships we found between major SQOL life domains and both depression and anxiety are crucial regarding BPD recovery, and indirectly confirm previous evidence linking depression in BPD with unfavourable outcomes such as impaired psychosocial and vocational functioning [1]. Bearing in mind that SQOL is adversely affected by impaired functioning [19], the negative links of depressive symptoms with SQOL in our study may also reflect a negative impact on psychosocial adjustment. Notably, SQOL measures (i.e., WHOQOL-BREF) have been used as a proxy of perceived functionality in BPD patients [46]. Moreover, our findings highlight the complex role of comorbidity in BPD, in particular concerning depression and anxiety. As previous evidence indicates [71], antidepressants are often ineffective for BPD patients’ depressive symptomatology. However, depression and anxiety may decrease with BPD improvement following successful types of psychotherapy [71,72,73], with some authors [1] arguing that depression is more a reflection of patients’ frustration with life and mainly an index of BPD severity rather than a comorbid disorder. The fact that in our study, the essential BPD features and the number of BPD criteria had no significant connections with SQOL above and beyond depression and anxiety may lend support to the above arguments, suggesting that depression and anxiety may be mediators of the relationship between BPD severity (manifested by aggravated and more numerous BPD features) and SQOL. That is, patients with severe BPD manifest greater levels of depression and anxiety, which in turn may exert a great impact on SQOL. However, this hypothesis should be examined in future studies with mediation analysis.

Another important finding in our study is that the co-occurrence of other PD(s) was also found to be related to SQOL in the critical domains of psychological and physical health. These results echo generally those of an earlier study on QOL in patients with various PDs [33], showing that the number of PDs together with the number of PD traits had the strongest detrimental effect on subjective well-being, highlighting the co-occurrence of more than one PD as an important predictor of SQOL. Furthermore, in our study, the significant relationships of SQOL with other PD(s) co-occurrence, together with the absence of substantial links of the number of BPD criteria, are in line with a prior study demonstrating that the major detrimental factor to SQOL is not the type of PD, but the number of PD diagnoses [34]. Furthermore, as with depression, the presence of other PD(s) is considered by many researchers [1,33] to be more of an indicator of BPD severity than an indication of another distinct comorbid disorder. In this regard, in the current study, both additional PDs and the number of BPD criteria were employed as disease severity measures. Our findings suggest that the co-existence of other PD(s) might be a significantly stronger determinant of SQOL than the number of BPD criteria, since it remained significant after depression and anxiety were entered into the models, whereas the number of BPD criteria did not exhibit any significant contribution to variance in SQOL domains. Thus, our second hypothesis regarding the part of additional PD(s) was confirmed, whereas the part regarding the number of BPD diagnostic criteria was disproved. Our results contrast with the study on SQOL of youths with BPD traits (subthreshold for BPD diagnosis) [38], in which the number of BPD traits was the second strongest predictor of SQOL after depression. This discrepancy in findings may suggest that the number of BPD criteria as a disease severity index may be a significant predictor of SQOL in sub-clinical populations with BPD traits, whereas in clinical BPD groups it is likely a weak predictor of SQOL compared to other psychopathological features of the disorder. Furthermore, our results indirectly confirm previous evidence indicating the number of BPD traits as a severity index with limited discriminating ability for patients who meet the DSM criteria for BPD compared to people with subthreshold BPD features [37].

Suicidality and self-harming behaviour were employed in the analysis, as they represent essential features of BPD. Our results indicated that suicidality and self-harming behaviour were not associated with any SQOL indices, above and beyond depression and anxiety. These results may be explained by the fact that high self-reported suicide risk, elevated depression, and anxious arousal are largely overlapping features in mental-health patients regardless of diagnosis, as has been documented in previous literature [74]. Importantly, this particular convergence of factors seems to be most effectively reflected by BPD, as was found in the study of Podlogar et al. [74]. In this respect, our findings suggest that depression and anxiety might fully mediate any possible association between suicidality /self-harming behaviour and SQOL. Additionally, alcohol and substance use disorders as manifestations of impulsivity were not significantly associated with any SQOL indices. These results may also suggest that depression and anxiety might fully mediate any potential link between alcohol/ substance use and SQOL.

Five out of six of our final models were significant, explaining a substantial portion of the variance in global QOL, global health, social relationships, physical and psychological health SQOL domains (between 11.8% and 35.4%). As could be expected, the psychopathological predictors, namely depression, occurrence of additional PD(s), state and trait anxiety explained the greatest proportion of variance in the psychological health SQOL domain. Environment was the only SQOL domain that was not significantly predicted by any variable in the analysis, probably due to the weak conceptual relevance between environment and psychopathology. Our regression models comprised the highest number of key BPD psychopathological predictors and exhibited higher predictive power compared to all previous reports in the field. Only one previous study reported models with similar predictive ability concerning global and domain-specific SQOL [19]. However, in addition to psychopathological variables, the predictive models in that study also included psychosocial functional predictors, which may have increased their explanatory power given the conceptual proximity of self-reported psychosocial functioning and SQOL [75,76,77].

Many clinical implications for SQOL enhancement result from our findings. Given the strong relationships of depression with SQOL in the most key life domains in BPD patients, regular and comprehensive assessment of depressive symptoms could be implemented in BPD services to facilitate early detection and treatment, which may ensure and enhance patients’ SQOL. Accordingly, as a considerable proportion of BPD patients experience anxiety and other PDs, [1], which both proved to have a significant negative relationship with SQOL, identifying and tackling these disorders through appropriate interventions could facilitate SQOL improvement. More specifically, given that (a) co-occurring disorders such as depression, anxiety and other PDs in BPD patients are rather severity indicators than comorbid disorders separate from the primary disease [1], and that (b) remission of BPD after successful treatment typically results in amelioration of the above associated psychopathology [71,72,73,78,79], evidence-based psychotherapeutic programs as TFP, MBT, DBT and ST could be deployed for the effective treatment of BPD, which will in turn achieve a crucial therapeutic goal: the enhancement of SQOL.

The study findings should be considered in light of some limitations. The design of the study was cross-sectional, meaning that the results cannot provide a causal interpretation, but an associative one. In addition, we did not exhaust neither all the clinical factors, especially other comorbidities (e.g., PTSD, eating disorders, obsessive compulsive disorder, psychotic disorders), as well as the use of medication, illness duration, and previous hospitalizations, nor all the sociodemographic data (income, employment, family status, living areas) that could be employed in the investigation of predictors of QOL in BPD. The included variables were chosen on a basis of clinical importance and reported relevance. We employed the number of BPD diagnostic criteria as an index of disorder severity to explore its association with SQOL. However, the index has a limited discriminating ability for patients who meet DSM diagnostic criteria for BPD compared to individuals with subthreshold BPD traits [37]. Perhaps it would be more appropriate to use a scale that assesses the severity of each BPD trait and the sum of individual trait severity scores (e.g., Borderline Personality Disorder Severity Index (BPDSI)) [80]. However, the standardization of such a tool in the Greek population has not yet been completed. Even though we had a broad age limit (18–56 years), the mean age of our sample was 29 years, which means that the findings are not representative for older age groups and cannot reflect on the course of the disorder. Moreover, the proportion of females was significantly larger than that of male participants, and thus gender differences in affect and self-esteem might have influenced the results. Finally, since our sample consisted of help-seeking outpatient individuals, the results cannot be generalized to clinically more severe BPD patients.

Consideration should also be given to the possibility of reverse causality between SQOL and emotional symptoms, i.e., low SQOL causing depression and anxiety. To our knowledge so far, no study has specifically addressed this direction of the association in mental health patients. According to WHO, systematic reviews have pointed out that interventions improving life satisfaction and quality of life have also significantly reduced affective clinical features [81]. In addition, living circumstances that provide favourable social, economic, physical and environmental conditions are linked to lower frequencies of common mental disorders, such as depression and anxiety [81]. In this respect, we can assume that poor SQOL per se might have a contribution to the emergence of depressive and/or anxiety symptoms. Thus, it is essential to further explore the possible bidirectionality of the association between SQOL and emotional symptoms.

Notably, other important non-clinical factors such as resilience and shame-proneness have been found to influence SQOL in BPD patients in parallel with psychopathological factors. Specifically, resilience has been indicated as a predictor of global SQOL in BPD patients before and after treatment [23], while poor ego-resiliency has been reported to negatively affect global SQOL in a BPD female population, with this relationship being mediated by both positive and negative affect [48]. Additionally, higher levels of shame were associated with lower global SQOL in women with BPD [82]. Future research on the determinants of SQOL in BPD could examine the complex relationship among resilience, shame-proneness and other non-clinical factors with BPD psychopathological dimensions to elucidate their impact on SQOL decline or improvement.

In conclusion, depression, anxiety and co-occurrence of other PD(s) emerged as the most powerful psychopathological dimensions associated with global and domain-specific SQOL in patients with BPD. In contrast, increasing numbers of BPD traits and central illness features, such as suicidality, self-harming behaviour, and impulsivity manifested through alcohol and substance use, did not exhibit significant associations with any SQOL dimension. These findings can be explained by the hypothesis that co-existing psychopathological syndromes in BPD patients, such as depression, anxiety and other PD(S), are more disease severity indices than comorbid disorders [1]. Hence, it is plausible that the aforementioned associated psychopathological manifestations serve as stronger severity indices than aggravated and more numerous BPD traits and might fully mediate the effect of BPD traits on SQOL. Future mediation analysis is needed to elaborate on this hypothesis, given that SQOL reflects psychosocial functioning in BPD patients, represents a marker of disease burden, and thus constitutes a crucial, recovery-oriented treatment goal and outcome.

## Figures and Tables

**Table 1:** Demographic, and clinical characteristics of the sample.

Characteristic	*n* (%)	Mean (SD)
Gender		
*Female*	100 (66.7)	
*Male*	30 (20.0)	
*Other*	20 (13.3)	
Age (years)		29.03 (9.42)
Education (years)		13.84 (2.05)
Family Status		
*Married*	7 (4.7)	
*Single*	132 (88.0)	
*Divorced*	11 (7.3)	
Employment		
*Employed*	57 (38.0)	
*Unemployed*	46 (30.7)	
*Student*	47 (31.3)	
Living area		
*Deprived*	21 (14.0)	
*Medium status*	102 (68.0)	
*Wealthy*	27 (18.0)	
Number of BPD criteria		6.35 (1.49)
Self-Harm Inventory		9.83 (4.45)
Suicide Risk Scale		9.73 (2.64)
Hospitalizations		0.38 (0.74)
BDI-II		31.67 (12.43)
STAI-State		54.45 (12.46)
STAI-Trait		60.87 (9.54)
Additional PD(s)	108 (72.0)	
AUD/SUD *	45 (30.0)	
Affective disorder	120 (80.0)	
Anxiety disorder	84 (56.0)	
Other disorder **	20 (13.3)	
WHOQOL-BREF		
*Physical Health*		11.43 (2.28)
*Psychological Health*		9.54 (2.92)
*Social Relationships*		10.61 (3.25)
*Environment*		12.64 (4.63)
*Overall QOL*		2.75 (0.94)
*Overall Health*		2.77 (1.11)

* Alcohol and Substance Use Disorder; ** Eating disorder, Obsessive Compulsive Disorder; BPD: Borderline Personality Disorder; PD: Personality Disorder; BDI II: Beck Depression Inventory-II; STAI: State and Trait Anxiety Inventory.

**Table 2:** The effect of demographic and clinical factors on WHOQOL-BREF domains in patients with BPD (*n* = 150). Hierarchical linear regressions.

	Physical Health			Psychological Health			Social Relationships			Environment			Overall QoL			Overall Health		
	Beta	*t*	*p*	Beta	*t*	*p*	Beta	*t*	*p*	Beta	*t*	*p*	Beta	*t*	*p*	Beta	*t*	*p*
***Step 1***																		
Gender (female)	0.08	1.09	0.275	0.07	0.98	0.331	0.00	0.03	0.977	−0.03	−0.36	0.718	0.02	0.28	0.776	−0.04	−0.50	0.618
Age	−0.08	−0.95	0.345	−0.06	−0.76	0.449	−0.24	−2.65	**0.009**	−0.09	−0.91	0.363	−0.05	−0.56	0.573	−0.14	−1.63	0.105
Education	0.13	1.65	0.102	0.13	1.77	0.079	−0.01	−0.16	0.869	−0.11	−1.18	0.238	0.09	1.07	0.286	0.18	2.18	**0.031**
*R^2^*	*0.028*			*0.044*			*0.044*			*0.016*			*0.009*			*0.030*		
Δ*F (3, 145), p*	*1.41*, *0.243*			*2.23*, *0.088*			*2.25*, *0.085*			*0.80*, *0.496*			*0.45*, *0.719*			*1.48*, *0.221*		
***Step 2***																		
Number of BPD criteria	−0.12	−1.21	0.226	0.00	0.05	0.962	−0.11	−1.10	0.271	0.16	1.48	0.141	−0.02	−0.20	0.844	−0.10	−1.04	0.301
Additional PD diagnosis	−0.19	−2.25	**0.026**	−0.16	−2.16	**0.032**	−0.03	−0.30	0.765	−0.02	−0.25	0.806	−0.06	−0.61	0.545	−0.08	−0.90	0.371
Self−Harm Inventory	0.06	0.58	0.561	−0.14	−1.63	0.104	0.10	0.95	0.345	−0.14	−1.25	0.212	0.11	1.03	0.304	−0.04	−0.41	0.680
Suicide Risk Scale	−0.14	−1.37	0.172 ^a^	0.04	0.45	0.651 ^a^	0.02	0.15	0.884	0.03	0.26	0.797	0.00	0.01	0.995	0.02	0.22	0.823
AUD/SUD	0.12	1.48	0.140	−0.10	−1.40	0.165	−0.03	−0.33	0.741	−0.08	−0.89	0.374	0.16	1.90	0.059	0.01	0.06	0.952
*R^2^*	*0.149*			*0.121*			*0.088*			*0.045*			*0.053*			*0.099*		
Δ*F (2, 143), p*	*3.96*, *0.002*			*4.18*, *0.001*			*1.34*, *0.252*			*0.84*, *0.523*			*1.29*, *0.271*			*2.15*, *0.063*		
***Step 3***																		
BDI−II	−0.43	−4.07	**<0.001**	−0.37	−3.87	**<0.001**	−0.33	−2.92	**0.004**	−0.08	−0.70	0.483	−0.26	−2.22	**0.028**	−0.17	−1.54	0.126
STAI−State	−0.12	−1.37	0.173	−0.16	−2.06	**0.041**	0.02	0.17	0.868	−0.08	−0.79	0.430	0.02	0.20	0.840	0.09	0.94	0.347
STAI−Trait	0.13	1.17	0.245	−0.21	−2.22	**0.028**	−0.04	−0.32	0.748	−0.04	−0.30	0.766	−0.10	−0.83	0.407	−0.33	−3.00	**0.003**
*R^2^*	*0.256*			*0.354*			*0.165*			*0.061*			*0.118*			*0.221*		
Δ*F (3, 140), p*	*6.58*, *<0.001*			*17.81*, *<0.001*			*4.23*, *0.007*			*0.78*, *0.507*			*3.35*, *0.021*			*7.13*, *<0.001*		

Note: Only Beta coefficients in the final model are displayed. R squares, F (df1, df2) change and *p* for change in F are reported for every step. ^a^ indicate that those variables significantly contribute to improvement of the adjusted R square in a previous step model, but lost their significance when variables in the further models were added. BPD: Borderline Personality Disorder; PD: Personality Disorder; BDI II: Beck Depression Inventory-II; STAI: State and Trait Anxiety Inventory; AUD/SUD: Alcohol and Substance Use Disorder.

## Data Availability

The data will be made available on request from the corresponding author.
